# Absence of constitutive EGF receptor activation in ovarian cancer cell lines.

**DOI:** 10.1038/bjc.1996.379

**Published:** 1996-08

**Authors:** C. Ottensmeier, L. Swanson, T. Strobel, B. Druker, J. Niloff, S. A. Cannistra

**Affiliations:** Division of Neoplastic Disease Mechanisms, Dana-Farber Cancer Institute, Boston MA 02115, USA.

## Abstract

**Images:**


British Journal of Cancer (1996) 74, 446-452
© 1996 Stockton Press All rights reserved 0007-0920/96 $12.00

### Absence of constitutive EGF receptor activation in ovarian cancer cell lines

C Ottensmeier[1], L Swanson[1], T Strobel[1], B Druker[2], J Niloff[3] and SA Cannistra[1]

[1]*Division of Neoplastic Disease Mechanisms, Dana-Farber Cancer Institute, 44 Binney Street, Boston MA 02115 and the Harvard Medical School, Boston, Massachusetts;* [2]*Oregon Health Sciences Center, 3181 SW Sam Jackson Park Road, L586, Portland OR 97201;* [3]*Beth Israel Hospital, 330 Brookline Ave., ST8M18, Boston MA 02215, USA.*

**Summary** Previous investigators have noted that certain ovarian cancer cell lines secrete and respond to transforming growth factor-α (TGF-α), suggesting that endogenous activation of the epidermal growth factor (EGF) receptor through autocrine or paracrine mechanisms might contribute to the proliferative response. In order to determine whether autocrine stimulation was partly responsible for the proliferative response in ovarian cancer, we investigated whether the EGF receptor expressed by ovarian cancer cell lines was constitutively activated as assessed by the presence of tyrosine phosphorylation. A specific anti-phosphotyrosine antibody was used in conjunction with an immunoblotting technique in order to detect EGF receptor phosphorylation in ovarian cancer cell lines in the absence and presence of exogenous EGF. The effects of neutralising anti-EGF receptor antibody on the proliferation of ovarian cancer cell lines was also examined. We found no evidence for constitutive tyrosine phosphorylation of the p170 EGF receptor in eight epithelial ovarian cancer cell lines tested, although each line demonstrated inducible phosphorylation in response to exogenous EGF. The absence of constitutive EGF receptor activation was also noted when cells were grown under high density conditions, thus excluding a role for membrane-bound EGF or TGF-α in this process. Media conditioned by five ovarian cancer cell lines, as well as malignant ascites obtained from 12 different ovarian cancer patients, were not capable of stimulating EGF receptor phosphorylation. Finally, the proliferation of ovarian cancer cell lines was not significantly inhibited in the presence of neutralising anti-EGF receptor antibody. These data suggest that EGF receptor activation through autocrine pathways is not a major mechanism for the growth of many ovarian cancer cell lines. Other pathways of signal transduction which bypass the requirement for EGF receptor activation may be important in the proliferation for ovarian cancer cells. Such EGF receptor-independent pathways may limit the effectiveness of strategies designed to inhibit ovarian cancer cell growth through disruption of EGF receptor function.

**Keywords:** ovarian cancer; epidermal growth factors receptor; tyrosine phosphorylation

Epithelial ovarian cancer is a highly lethal disease which spreads extensively throughout the abdominal cavity. Factors which predict for poor outcome include advanced stage, older age, high tumour grade, amplification of the c-*neu* proto-oncogene and overexpression of the epidermal growth factor (EGF) receptor (Slamon *et al.*, 1989; Berchuck *et al.*, 1991; Scambia *et al.*, 1992; Cannistra, 1993). The association between EGF receptor expression and poor prognosis has raised the possibility that this receptor may be involved in the proliferative response of ovarian cancer cells *in vivo* through autocrine or paracrine mechanisms. In this regard, many ovarian cancer cell lines as well as cells from fresh ovarian tumours respond to exogenous EGF *in vitro*, and some ovarian cancer cell lines express mRNA and protein for transforming growth factor-alpha (TGF-α), a known ligand for the EGF receptor (Rodriguez *et al.*, 1991; Crew *et al.*, 1992; Stromberg *et al.*, 1992; Zhou and Leung, 1992; Morishige *et al.*, 1993). Some investigators have also shown that neutralising anti-TGF-α antibody is capable of partly inhibiting proliferation of certain ovarian cancer cells lines *in vitro*, suggesting involvement of TGF-alpha and the EGF receptor in an autocrine loop (Stromberg *et al.*, 1992; Morishige *et al.*, 1993).

The EGF receptor is widely expressed on several types of epithelial cells, including the normal ovarian surface epithelium (NOSE) which gives rise to most cases of ovarian cancer (Bast *et al.*, 1992). This receptor is a membrane tyrosine kinase which characteristically forms homodimers after ligand binding to either EGF or TGF-α (Ullrich and Schlessinger, 1990). Homodimerisation results in stimulation of tyrosine kinase activity and autophosphorylation of several tyrosine moieties contained within the receptor's cytoplasmic domain. Since tyrosine phosphorylation is critical to EGF receptor function, detection of phosphorylated tyrosine moieties provides an accurate assessment of this receptor's activation state (Carpenter and Cohen *et al.*, 1990; Ullrich and Schlessinger, 1990).

The expression of EGF receptors by ovarian cancer cells and the ability of these cells to respond to EGF *in vitro* provides only circumstantial evidence that this signal transduction pathway is involved in the proliferative response. Therefore, the purpose of this study was to define more accurately the activation status of the EGF receptor in ovarian cancer cells by performing immunoblotting with a specific anti-phosphotyrosine antibody. Our results demonstrate that the EGF receptor is not constitutively activated in many ovarian cancer cell lines, and that the proliferation of ovarian cancer cells is not significantly inhibited in the presence of neutralising anti-EGF receptor antibody. The implications of these observations for ovarian cancer pathogenesis are discussed.

### Materials and methods

### *Reagents*

Immunoblotting was performed using a previously described murine anti-phosphotyrosine (P-Tyr) monoclonal antibody (MAb) developed by one of us (BD). This antibody is specific for phosphotyrosine, as demonstrated by complete elimination of immunoreactive bands with the addition of 1 mmol $l^{-1}$ phosphotyrosine, and it does not recognise phosphoserine or phosphothreonine moieties (Kanakura *et al.*, 1990). Murine anti-EGF receptor (anti-EGF-R) antibody (Clone ZO25) used for immunoprecipitation and immunoblotting was purchased from Zymed (So. San Francisco, CA, USA). Neutralising murine anti-EGF-R antibody (clone 225)

Correspondence: SA Cannistra, Division of Neoplastic Disease Mechanisms, Dana-Farber Cancer Institute, 44 Binney Street, Boston MA 02115, USA
Received 10 November 1995; revised 16 February 1996; accepted 27 February 1996

used for inhibition of cell line proliferation was purchased from Oncogene Science (Uniondale, NY, USA). Recombinant human EGF (rhEGF) was purchased from Amgen (Thousand Oaks, CA, USA), and rhTGF-$\alpha$ was purchased from Collaborative Biomedical Products (Bedford, MA, USA). Insulin, transferrin and cholesterol used for preparation of serum-free medium were purchased from Sigma Chemical Company (St Louis, MO, USA).

*Source of cells*

Ovarian epithelial carcinoma cell lines used in this study include CAOV-3, SKOV-3, OVCAR-3 and SW626 and were obtained from the American Type Culture Collection (ATCC) (Rockville, MD, USA). These cells were cultured in Iscove's modified Dulbecco's minimal essential medium (IMDMEM) (Gibco BRL, Grand Island, NY, USA) supplemented with 20% fetal calf serum (FCS) (HyClone, Logan, UT, USA). An additional cell line was developed in our laboratory from the malignant ascites of a patient, designated unique patient number 36 (UPN36), with moderately well-differentiated serous papillary carcinoma of the ovary as previously described (Cannistra *et al.*, 1993). This line is called UPN36T and was derived by injecting $100 \times 10^6$ ascites cells intraperitoneally into a female Swiss Nu/Nu athymic nude mouse (Taconic, Germantown, NY, USA) and isolating a peritoneal implant which contained immortalised tumour cells capable of continuous *in vitro* growth in 20% FCS/IMDMEM. Three other lines (UPN11, UPN13 and UPN21) developed by us were derived from *in vitro* culture of cells obtained from malignant ascites. All ovarian cancer cell lines have been passaged for over 1 year in IMDMEM containing 10–20% FCS without the addition of exogenous growth factors, and they express both keratin and vimentin as assessed by immunoperoxidase staining. The A431 squamous cell carcinoma cell line was used as a positive control for constitutive EGF receptor activation (Van de Vijver *et al.*, 1991) and was purchased from the ATCC. Malignant ascites was obtained from ovarian cancer patients undergoing therapeutic paracentesis for the relief of abdominal distension. Tissue procurement was approved by the Institutional Review Board of the Dana-Farber Cancer Institute.

*Immunoblotting*

Cells were grown at subconfluent density for 18 h in either serum-free media (SFM)(IMDMEM containing 1 $\mu$g ml$^{-1}$ insulin, 5 $\mu$M transferrin, 10 $\mu$g ml$^{-1}$ cholesterol) or 10% FCS before EGF stimulation. After exposure to either media alone or EGF (10 ng ml$^{-1}$) for 10 min, cells were lysed for 30 min at 4°C in lysis buffer (1% NP40, Tris 50 mM, sodium chloride 150 mM) containing 100 mM phenylemethylsulphonyl fluoride (PMSF), 0.135 trypsin-inhibitory units of aprotinin and 40 $\mu$M leupeptin and 5 mM sodium orthovanadate (Sigma). Proteins were resolved (250 $\mu$g per lane) by one-dimensional SDS-polyacrylamide gel electrophoresis (SDS-PAGE) as previously described, followed by transfer onto a 0.2 $\mu$M nitrocellulose filter (Schleicher and Schuell, Keene, NH, USA) in transfer buffer at 0.1 amp overnight 4°C (Kanakura *et al.*, 1990). After transfer, residual binding sites were blocked by incubating the membrane in Tris-buffered saline (TBS) containing 1% gelatin (BioRad Laboratories, Melville, NY, USA) for 1 h at room temperature (RT). The blots were then washed in TBS with 0.05% Tween 20 (TBST) and incubated for at least 4 h at RT with the primary antibody (either anti-P-Tyr or anti-EGF receptor MAb, 1.5–2 $\mu$g ml$^{-1}$ in TBST). The blots were then washed four times in TBST, followed by incubation in a 1:2000 dilution of anti-mouse IgG conjugated to alkaline phosphatase (Promega, Madison, WI, USA) in TBST for 2 h at RT. After three additional washes in TBST, the blot was placed in a buffer containing 100 mmol l$^{-1}$ Tris-HCl, pH 9.5, 100 mmol m$^{-1}$ sodium chloride, 5 mmol l$^{-1}$ magnesium chloride, 330 $\mu$g ml$^{-1}$ nitro blue tetrazolium (NBT) and 150 $\mu$g ml$^{-1}$ 5-bromo-4-chloro-3-indolyl phosphate (BICP) for 10–30 min. The enzymatic colour reaction was stopped by rinsing the filters in deionised water.

*Immunoprecipitation*

For some experiments, immunoprecipitation of the EGF receptor was performed before immunoblot analysis. Briefly, $7.5 \times 10^6$ cells were suspended in 1 ml lysis buffer for 30 min at 4°C. After lysis, the solubilised fraction was obtained by centrifugation (14 000 r.p.m. × 30 min) to remove insoluble debris, followed by preclearing with 50 $\mu$l of a 1:1 slurry of lysis buffer and Protein A-Sepharose beads (type CL-4B, Pharmacia, Piscataway, NJ, USA) precoated with polyclonal rabbit anti-mouse immunoglobulin (RaM Ig, Dako). After centrifugation, the precleared supernatant was incubated with either an isotype-identical irrelevant antibody (D144) or with anti-EGF receptor antibody and rabbit anti-mouse Ig-coated Protein A-Sepharose beads for 15 h at 4°C, followed by pelleting and washing three times in lysis buffer. The immunoprecipitate was boiled for 5 min at 100°C in non-reducing conditions and analysed by SDS-PAGE, followed by protein transfer and immunoblot analysis as described above.

*Proliferation assay*

The effects of EGF or anti-EGF-R antibody on the proliferation of ovarian cancer cell lines were assessed by measuring the cleavage of MTT (diemthylthiazol-diphenyl tetrazolium bromide, Sigma) to formazan as previously described (Mosmann, 1983). Briefly, cells ($2.5 \times 10^3$ per well) were added in quadruplicate to 96-well microtitre plates in a total volume of 100 $\mu$l of IMDMEM containing 10% FCS and allowed to incubate at 37°C for a total of 120 h. During the last 4 h of incubation, 10 $\mu$l of MTT (5 mg ml$^{-1}$ in PBS) was added, followed by the addition of 100 $\mu$l of 0.04 N hydrochloric acid in isopropanol to dissolve the formazan. After mixing, the optical density of each well was measured on an ELISA plate reader at a wavelength of 590 nm. Control cultures included incubation of cells in anti-CD44 antibody (clone 515), which is an IgG1 murine monoclonal antibody which binds to the ovarian cancer cells used in this study but does not significantly affect their proliferation (Cannistra *et al.*, 1993). Specific optical density (OD) was defined as the OD of the treatment group containing cells minus the OD of wells containing media without cells. Data were expressed as the stimulation index, which is a ratio of the specific OD of the treatment group divided by the specific OD of cells grown in the presence of control antibody (anti-CD44 antibody).

*Statistical analysis*

Data are expressed as mean ± standard error of the mean (s.e.m.) where appropriate. Significance levels for comparison of stimulation indices between treatment groups were determined using the two-sided Student's *t*-test for unpaired samples.

### Results

*Status of EGF receptor tyrosine phosphorylation in ovarian cancer cell lines*

In order to assess the phosphorylation status of the EGF receptor in ovarian cancer cell lines, we performed immunoblotting of a variety of cell lysates using a murine anti-phosphotyrosine antibody. In pilot studies using the CAOV-3 line, we have shown that this technique is capable of detecting inducible phosphorylation of the p170 kDa EGF receptor in the presence of $\geqslant 0.5$ ng ml$^{-1}$ EGF after a 10 min exposure at 37°C, with maximum phosphorylation observed at a dose of 10 ng ml$^{-1}$ EGF. The results of a typical immunoblotting analysis for seven ovarian cancer lines stimulated with or without 10 ng ml$^{-1}$ EGF (10 min at 37°C) are shown in Figure 1a and b. In these experiments, cells were split the night before and grown at subconfluent density for 18 h in SFM before immunoblotting. There was no evidence of EGF receptor phosphorylation in any cell line in the absence of exogenous EGF stimulation. In contrast, a phosphotyrosine-containing molecule at 170 kDa which was consistent with the EGF receptor (Carpenter and Cohen, 1990; Ullrich and Schlessinger, 1990) was observed for each cell line in the presence of EGF. Other less prominent EGF-inducible bands appear in the ranges of 66, 52 and 44/42 kDa. The species at 66 and 52 kDa are consistent with the known molecular masses of the SHC family of proteins (Pelicci *et al.*, 1992), and the p44/42 species are consistent with the known molecular masses of MAP kinase (Wu *et al.*, 1991). A similar pattern of inducible EGF receptor phosphorylation was observed for the SKOV-3 cell line (data not shown). Results were identical for cells grown in 20% FCS/IMDMEM (instead of SFM) for 18 h before immunoblotting. Finally, in order to ensure that the immunoblotting technique used in this study was capable of detecting constitutive tyrosine phosphorylation of the EGF-R, we determined the status of basal EGF-R phosphorylation in A431 cells. This cell line has been shown previously to express activated EGF-R through autocrine secretion of TGF-α (Van de Vijver *et al.*, 1991). As shown in Figure 1c, a constitutively phosphorylated 170 kDa protein is observed in A431 cells in the absence of EGF, although phosphorylation is upregulated in the presence of exogenous ligand. In comparison, there was no evidence of constitutive EGF-R activation in CAOV-3, UPN36T or SW626 cells.

### *Identification of p170 as the EGF receptor*

In order to confirm that p170 was identical to the EGF receptor, we first treated CAOV-3 cells with or without EGF as described, followed by immunoprecipitation of cell lysates with an anti-EGF receptor (anti-EGF-R) antibody. Each immunoprecipitate was divided into two equal aliquots which were then separately resolved by SDS-PAGE for subsequent immunoblotting with either anti-EGF-R or anti-phosphotyrosine antibody (Figures 2a and b). Whole cell lysates were also loaded to serve as a control for the presence of p170. Figure 2a shows the results of immunoblotting with the anti-EGF-R antibody, demonstrating an EGF receptor species at 170 kDa in the whole cell lysate groups (lanes A and D) and in the groups immunoprecipitated with anti-EGF receptor antibody (lanes C and F). As expected, the EGF receptor is not observed in the groups immunoprecipitated with control antibody (anti-D144, lanes B and E). When these same lysates are immunoblotted with anti-phosphotyrosine antibody as shown in Figure 2b, no reactivity is observed for either whole cell lysates or anti-EGF receptor immunoprecipitates of cells treated with media alone (lanes A and C). In contrast, there is strong expression of the p170 phosphotyrosine in both whole cell lysates as well as the EGF receptor immunoprecipitates in the presence of EGF (lanes D and F). These data demonstrate that the p170 band observed in whole cell lysates is identical to the EGF receptor.

### *The effects of conditioned media and ascites on EGF receptor phosphorylation*

The majority of immunoblotting experiments performed in this study involved ovarian cancer cell lines which were grown for 18 h at a subconfluent density. We considered the possibility that these conditions might result in endogenous levels of secreted EGF or TGF-α which are insufficient to produce EGF receptor activation, perhaps leading to falsely negative results. In order to exclude this possibility, we first conditioned media for 48 h in the presence of 20% FCS using a variety of ovarian cancer cell lines at near confluence.

**Figure 1** Status of p170 tyrosine phosphorylation in ovarian cancer cell lines. Cells were incubated in SFM for 18 h and stimulated with either SFM alone or with EGF (10 ng ml$^{-1}$) for 10 min before lysis. Lysates were resolved by SDS-PAGE under reducing conditions, transferred to nitrocellulose and developed using an anti-phosphotyrosine antibody. The EGF receptor has an expected band molecular mass of 170 kDa and is not observed in unstimulated cells. In contrast, a phosphotyrosine protein at 170 kDa is observed upon EGF exposure, associated with additional bands at 66, 52, 44 and 42 kDa. Similar results were obtained for cells incubated in 20% FCS/IMDMEM instead of SFM. Data shown are representative results from one of three separate experiments. (**a**) Immunoblot using lysates from CAOV-3, UPN13, UPN21 and UPN36T cell lines. (**b**) Immunoblot using lysates from the OVCAR-3, SW626 and UPN11 cell lines. (**c**) Immunoblot using lysates from A431 cells, demonstrating constitutive tyrosine phosphorylation of the EGF-R in the absence of exogenous EGF (in contrast to CAOV-3, UPN36T and SW626 cells).

Medium containing 20% FCS was used to ensure maximum growth of the culture over a more prolonged incubation period, as cells exposed to SFM over 48 h acquire a non-adherent morphology associated with loss of viability. As stated above, the use of 20% FCS-containing medium by itself was not capable of stimulating EGF receptor phosphorylation, suggesting that the final concentration of EGF in this medium was less than 0.5 ng $ml^{-1}$. After 48 h, conditioned media were then used to stimulate EGF receptor phosphorylation in CAOV-3 cells as assessed by immunoblotting. As shown in Figure 3, none of the conditioned media (CM) from the five cell lines tested was capable of inducing EGF receptor phosphorylation (10 min exposure of straight CM at 37°C before CAOV-3 cell lysis). Similar results were obtained by using media conditioned by the same ovarian cancer cell lines for up to 96 h. In addition to conditioned media, we also studied ascites samples from 12 separate ovarian cancer patients in order to determine whether they contained physiologically relevant levels of either EGF or TGF-α. As shown in Figure 4, none of the ascites samples were capable of inducing p170 tyrosine phosphorylation in CAOV-3 cells. Co-incubation of EGF (10 ng $ml^{-1}$) with 20% FCS/IMDMEM or ascites for 48 h did not diminish its ability to induce EGF receptor phosphorylation, suggesting that growth factor degradation was not responsible for the negative results shown in Figures 3 and 4. Finally, we considered the possibility that EGF or TGF-α may be presented to cells in a membrane-bound form, which would require cell–cell contact for EGF receptor stimulation. Therefore, CAOV-3 cells were grown in 20% FCS/IMDMEM for either 24 h (subconfluent) or 96 h (confluent) before assessing EGF receptor phosphorylation status. As shown in Figure 5, there was no evidence of constitutive EGF receptor phosphorylation for either subconfluent or confluent cells. The fact that p170 phosphorylation could be induced in confluent cells by EGF (Figure 5) demonstrates the presence of functional receptors at the cell surface and excludes the possibility that receptor down-regulation is responsible for the lack of constitutive activation at 96 h.

**Figure 2** Immunoprecipitation of the EGF receptor in CAOV-3 cells. CAOV-3 cells were treated with either media (SFM) alone or with EGF as described previously, followed by lysis and immunoprecipitation using a control antibody (anti-D144) or an isotype-identical murine monoclonal antibody reactive with the EGF receptor (anti-EGF-R). Whole cell lysates (which were not immunoprecipitated) were also saved for SDS-PAGE analysis. The immunoprecipitates were divided into two equal fractions and run on two separate gels for immunoblotting with either anti-EGF-R (**a**) or with anti-phosphotyrosine antibody (**b**). (**a**) Immunoblotting with an anti-EGF-R antibody reveals a band at 170 kDa corresponding to the EGF receptor in both whole cell lysates and anti-EGF-R immunoprecipitates (lanes A, C, D and F). The 170 kDa band was not observed in the immunoprecipitates using control antibody (lanes B and E). The band at ≈100 kDa is non-specific. (**b**) Immunoblotting with anti-phosphotyrosine antibody reveals an EGF-inducible band at 170 kDa in whole cell lysates (lane D) which is identified as the EGF receptor in lane F.

**Figure 3** Effects of media conditioned by ovarian cancer cell lines on EGF receptor tyrosine phosphorylation in CAOV-3 cells. Conditioned media (CM) were generated by culturing a variety of ovarian cancer cell lines in 20% FCS/IMDMEM for 48 h. CAOV-3 cells were subsequently exposed to either SFM, EGF (10 ng $ml^{-1}$), 20% FCS/IMDMEM (without conditioning) or to CM from the indicated lines for 10 min. Immunoblotting with anti-phosphotyrosine antibody was then performed, revealing inducible phosphorylation of p170 in only EGF-treated cells.

*Effects of anti-EGF receptor neutralising antibody on the proliferation of ovarian cancer cell lines*

These data suggest that significant levels of constitutive EGF receptor activation are not responsible for the proliferation of the ovarian cancer cell lines used in this study. However, we also considered the possibility that the sensitivity of immunoblot analysis might not be sufficient to exclude definitively an autocrine pathway of EGF receptor activation mediated through EGF or TGF-α secretion. In order to determine whether external activation of the EGF receptor might be partly responsible for the proliferation of ovarian cancer cell lines, we incubated cells in the presence of either EGF or anti-EGF-R antibody for 120 h, followed by assessment of proliferation by the MTT assay as described. As shown in Table I, the A431 squamous cell carcinoma cell line was significantly inhibited by 10 $\mu$g ml$^{-1}$ of anti-EGF-R antibody, with a stimulation index of 0.58 compared with control antibody (anti-CD44) ($P$=0.001). EGF (10 ng ml$^{-1}$) resulted in an inhibitory effect in A431 cells, consistent with the known ability of relatively high concentrations of this factor to induce apoptosis in this cell line (Gulli *et al.*, 1995). The inhibitory effect of EGF on A431 cells was blocked by anti-EGF-R antibody (10 $\mu$g ml$^{-1}$), thus demonstrating the specificity of this antibody for the EGF receptor (data not shown). In each of five ovarian cancer lines tested, anti-EGF-R antibody resulted in a minor inhibitory effect, with stimulation indices ranging from 0.86 to 0.94. None of these effects was statistically significant or reached the magnitude of the effect observed in A431 cells. Finally, exogenous EGF was observed to induce a statistically significant, albeit modest, proliferative effect in three out of five lines (SW626, CAOV-3 and SKOV-3), whereas it exerted no effects in the UPN36T or OVCAR-3 lines (despite the ability of EGF to induce receptor phosphorylation in these lines as shown in Figure 1). EGF was not capable of stimulating proliferation in any cell line at a concentration below 1.0 ng ml$^{-1}$. A similar pattern of growth stimulation was observed with the use of TGF-α between a range of 1.0–10 ng ml$^{-1}$ (data not shown).

**Table I** Effects of anti-EGF receptor antibody on the proliferation of ovarian cancer cell lines

| | *Stimulation index*[a] | |
|---|---|---|
| *Cell line*[b] | *Anti-EGF-R antibody*[c] | *EGF (10 ng ml$^{-1}$)* |
| A431 | 0.58±0.03 ($P$ = 0.001)[d] | 0.79±0.35 ($P$ = 0.004) |
| UPN36T | 0.88±0.07 ($P$ = 0.14) | 1.04±0.16 ($P$ = 0.73) |
| SKOV-3 | 0.89±0.05 ($P$ = 0.06) | 1.19±0.06 ($P$ = 0.016)[e] |
| SW626 | 0.94±0.04 ($P$ = 0.15) | 1.28±0.01 ($P$ = 0.001) |
| CAOV-3 | 0.91±0.06 ($P$ = 0.16) | 1.23±0.06 ($P$ = 0.02) |
| OVCAR-3 | 0.86±0.07 ($P$ = 0.08) | 1.01±0.12 ($P$ = 0.18) |

[a]Proliferation was assessed by the MTT assay after 120 h of incubation as described in the text. The stimulation index is a ratio of the specific optical density (OD) of the treatment group divided by the specific OD of control cells grown in the presence of an irrelevant antibody which has no effect on proliferation (anti-CD44 antibody, 10 $\mu$g ml$^{-1}$). [b]The A431 cervical cancer cell line was used as a positive control for the effects of anti-EGF receptor (anti-EGF-R) antibody, which is known to block the component of A431 proliferation due to autocrine secretion of TGF-α. The remaining cell lines are ovarian in origin as described in the text. [c]Anti-EGF-R antibody was used at a final concentration of 10 $\mu$g ml$^{-1}$, since this dose was found to produce a maximal inhibitory effect on A431 proliferation in pilot experiments. [d]Data are presented as mean±standard error of the mean (s.e.m.) stimulation index of three separate experiments. $P$-values were determined by the Student's $t$-test for unpaired samples. [e]Stimulation of SKOV-3, SW626, and CAOV-3 was maximal at 10 ng ml$^{-1}$ of exogenous EGF and was not observed at EGF concentrations below 1.0 ng ml$^{-1}$. A similar pattern of growth stimulation was observed with the use of TGF-α between a range of 1.0–10 ng ml$^{-1}$ (data not shown).

**Figure 4** Malignant ascitic fluid from ovarian cancer patients does not induce EGF receptor tyrosine phosphorylation in CAOV-3 cells. Ascites from 12 separate patients with newly diagnosed ovarian cancer was collected in heparin (100 U ml$^{-1}$), followed by centrifugation and collected of the cell-free fraction which was used in these experiments. CAOV-3 cells were exposed to either SFM, EGF (10 ng ml$^{-1}$) or to undiluted ascites as indicated for 10 min at 37°C. None of the ascites samples was capable of inducing tyrosine phosphorylation of p170 in CAOV-3 cells.

**Figure 5** Effects of CAOV-3 cell density on EGF receptor tyrosine phosphorylation. CAOV-3 cells were plated at a subconfluent density (0.1 × 10$^6$ ml$^{-1}$ 20% FCS/IMDMEM) and allowed to grow for either 24 or 96 h before immunoblot analysis as described. At 24 h the cells were non-confluent, whereas a confluent monolayer was present at 96 h. There was no evidence of constitutive p170 tyrosine phosphorylation under confluent or non-confluent conditions, although phosphorylation was inducible by a 10 min exposure to exogenous EGF (10 ng ml$^{-1}$).

### Discussion

The contribution of an autocrine or paracrine loop to the growth of epithelial ovarian cancer cells has been difficult to determine. Many human ovarian cancer cell lines are responsive to EGF but are not dependent upon addition of this growth factor for *in vitro* propagation (Rodriguez *et al.*, 1991; Crew *et al.*, 1992; Zhou and Leung, 1992). This observation has raised the possibility that autonomous growth of ovarian cancer cells may be mediated through either autocrine or paracrine secretion of EGF receptor ligands (Morishige *et al.*, 1993; Stromberg *et al.*, 1992). Alternatively, the growth of malignant ovarian epithelial cells could occur through the activation of signal transduction pathways which are independent of the EGF receptors. The purpose of the present study was to distinguish between these two possibilities by assessing the activation status of the EGF receptor expressed by a variety of ovarian cancer cell lines. By performing immunoblotting with a specific anti-phosphotyrosine antibody, we have shown that each of the eight ovarian cancer cell lines used in this study expressed EGF receptors, as manifested by inducible tyrosine phosphorylation of p170 after EGF exposure. However, none of these lines demonstrated constitutive activation of the EGF receptor in the absence of ligand. Media conditioned by these lines for up to 48 h also failed to stimulate receptor phosphorylation, as did malignant ascites samples from 12

different patients. There was no evidence for receptor activation upon cell–cell contact, excluding an important role of membrane-bound EGF or TGF-α in this process. Finally, anti-EGF-R antibody did not exert a significant inhibitory effect on the proliferation of ovarian cancer cell lines tested in this study. Taken together, these data suggest that constitutive activation of the EGF receptor, either through autocrine or paracrine mechanisms, is not a common feature of many ovarian cancer cell lines.

The immunoblotting assay used in these experiments was ideally suited to determining the role of constitutive EGF receptor activation in ovarian cancer for several reasons. This assay detected an inducible tyrosine phosphoprotein of 170 kDa which was shown to be specific for the EGF receptor by immunoprecipitation. In addition, p170 was phosphorylated by as little as 0.5 ng $ml^{-1}$ EGF, a concentration which typically produces an absent or low level proliferative response in the ovarian cancer cell lines used in this study (data not shown). It is unlikely, therefore, that biologically significant amounts of EGF receptor ligand present in conditioned medium or ascites would be missed in these experiments. Although it is certainly possible that more sensitive assays of EGF or TGF-α measurement would reveal the presence of these cytokines in conditioned medium or ascites, the biological significance of these levels in the absence of a significant effect on EGF receptor phosphorylation is difficult to determine. For instance, the use of radioimmunoassay has revealed low level expression of TGF-α in the range of 0.016–0.197 ng $ml^{-1}$ in many epithelial ovarian cancer cell lines, including OVCAR-3 and SKOV-3 (Stromberg *et al.*, 1992). The fact that we were unable to detect growth stimulation at these TGF-α (or EGF) concentrations suggests that this level of growth factor secretion may not be relevant to the *in vitro* proliferation of the ovarian cancer cell lines used in this study. Also, constitutive tyrosine phosphorylation of the EGF receptor in A431 cells was easily detected by immunoblotting (Figure 1c), suggesting that this assay should be capable of demonstrating this phenomenon in the other cell lines used in this study. It is important to note, however, that A431 cells have been shown to overexpress constitutively phosphorylated EGF receptors dramatically (Gulli *et al.*, 1995), raising the possibility that the immunoblot assay used in this study may not always be capable of detecting constitutive phosphorylation of lower numbers of receptors typical of ovarian cancer cell lines (Rodriguez *et al.*, 1991). Finally, it is known that constitutive activation of EGF receptor function may occur through at least three distinct pathways, including autocrine stimulation of the receptor by secreted ligands present in the extracellular space (Di Marco *et al.*, 1989; Stromberg *et al.*, 1992; Morishige *et al.*, 1993, autocrine stimulation by non-secreted ligands present in the cytoplasmic compartment (Keating and Williams, 1988), and upregulation of function owing to EGF receptor truncation (Khazaie *et al.*, 1988). Since tyrosine phosphorylation is a common feature of each of these pathways, the immunoblotting technique has the additional advantage of allowing the detection of activated EGF receptors regardless of their mechanism of stimulation.

Although all ovarian cancer cell lines used in this study demonstrated inducible tyrosine phosphorylation of the EGF receptor, two of the five lines tested failed to show a proliferative response in the presence of this growth factor (UPN36T) and OVCAR-3). This observation is consistent with the experience of other investigators (Rodriguez *et al.*, 1991), who have noted lack of EGF responsiveness despite the presence of adequate EGF receptor surface expression. The fact that EGF receptor activation is not always associated with a proliferative response, coupled with the observation that this receptor is not constitutively phosphorylated, suggests that other pathways of signal transduction may be important in ovarian cancer cell growth. In this regard, it is interesting to note that Mills *et al.* (1988, 1990) have characterised a soluble growth factor present in the malignant ascites of ovarian cancer patients which is capable of inducing ovarian cancer cell proliferation. This factor appears to be distinct from EGF by its ability to induce increases in intracellular calcium when added to ovarian cancer cells *in vitro* (Mills *et al.*, 1988). In addition, several other EGF-independent pathways of signal transduction have been identified, including activation of the c-*neu* proto-oncogene, a protein which is capable of inducing malignant transformation *in vitro* and which is overexpressed in over 20% of ovarian cancer specimens (Di Fiore *et al.*, 1987; Hudziak *et al.*, 1987; Slamon *et al.*, 1989). Abnormalities of tumour-suppressor proteins such as p53, which is mutated in over 50% of ovarian cancer specimens (Kupryjanczyk *et al.*, 1993; Milner *et al.*, 1993; Teneriello *et al.*, 1993), may also be a critical component of disordered growth regulation in this disease. It is possible that such EGF receptor-independent pathways may limit the effectiveness of strategies designed to inhibit ovarian cancer cell growth through disruption of EGF receptor function. Although we cannot exclude a possible contributory role of EGF receptor-mediated proliferation in ovarian cancer pathogenesis, our data suggest that constitutive activation of the EGF receptor may not be an important component of the proliferative response in at least some cases of ovarian cancer.

**Acknowledgements**
SAC is supported in part by Public Health Service grant CA 60670. CO is a grant recipient of the Deutsche Forschungsgemeinschaft.

### References

BAST RC Jr, JACOBS I AND BERCHUCK A. (1992). Malignant transformation of ovarian epithelium. *J. Natl Cancer Inst.*, **52**, 5322–5328.

BERCHUCK A, RODRIGUEZ GC, KAMEL A, DODGE RK, SOPER JT, CLARKE-PEARSON DL AND BAST RC Jr. (1991). Epidermal growth factor receptor expression in normal ovarian epithelium and ovarian cancer. I. Correlation of receptor expression with prognostic factors in patients with ovarian cancer. *Am. J. Obstet. Gynecol.*, **164**, 669–674.

CANNISTRA SA. (1993). Cancer of the ovary. *N. Engl. J. Med.*, **329**, 1550–1559.

CANNISTRA SA, KANSAS GS, NILOFF J, DEFRANZO B, KIM Y AND OTTENSMEIER C. (1993). Binding of ovarian cancer cells to peritoneal mesothelium *in vitro* is partly mediated by CD44H. *Cancer Res.*, **53**, 3830–3838.

CARPENTER G AND COHEN S. (1990). Epidermal growth factor. *J. Biol. Chem.*, **265**, 7709–7712.

CREW AJ, LANGDON SP, MILLER EP AND MILLER WR. (1992). Mitogenic effects of epidermal growth factor and transforming growth factor-alpha on EGF-receptor positive human ovarian cancer cell lines. *Eur. J. Cancer*, **28**, 337–341.

DI FIORE PP, PIERCE JH, KRAUS MH, SEGATTO O, KING CR AND AARONSON SA. (1987). erbB-2 is a potent oncogene when overexpressed in NIH/3T3 cells. *Science*, **237**, 178–182.

DI MARCO E, PIERCE JH, FLEMING TP, KRAUS MH, MOLLOY CJ, AARONSON SA, AND DIFIORE PP. (1989). Autocrine intereaction between TGF-a and the EGF receptor: Quantitative requirements for induction of the malignant phenotype. *Oncogene*, **4**, 831–838.

GULLI LF, KIM HR, PALMER K AND REDDY KB. (1995). Apoptosis induced by the epidermal growth factor is rescued by tyrosine kinase inhibitor in A431 cells. *Proc. Am. Assoc. Cancer Res.*, **36**, 10.

HUDZIAK RM, SCHLESSINGER J AND ULLRICH A. (1987). Increased expression of the putative growth factor receptor p185$^{HER2}$ causes transformation and tumorigenesis of NIH 3T3 cells. *Proc Natl Acad. Sci. USA*, **84,** 7159-7163.

KANAKURA Y, DRUKER B, CANNISTRA SA, FURUKAWA Y, TORIMOTO Y AND GRIFFIN JD. (1990). Signal transduction of the human granulocyte-macrophase colony-stimulating factor and interleukin-3 receptors involves tyrosine phosphorylation of a common set of cytoplasmic proteins. *Blood*, **76,** 706-715.

KEATING MT AND WILLIAMS LT. (1988). Autocrine stimulation of intracellular PDGF receptors in v-sis-transformed cells. *Science*, **239,** 914-916.

KHAZAIE K, DULI TJ, GRAF T, SCHLESSINGER J, ULLRICH A, BEUG H AND VEENSTROM B. (1988). Truncation of the human EGF receptor leads to differential transforming potentials in primary avian fibroblasts and erythroblasts. *EMBO J.*, **7,** 3061-3071.

KUPRYJANCZYK V, THOR AD, BEAUCHAMP R, MERRITT V, EDGERTON SM, BELL DA AND YANDELL DW. (1993). p53 gene mutations and protein accumulation in human ovarian cancer. *Proc. Natl Acad. Sci. USA*, **90,** 4961-4965.

MILLS GB, MAY C, MCGILL M, ROIFMAN CM AND MELLORS A. (1988). A putative new growth factor in ascitic fluid from ovarian cancer patients: identification, characterization, and mechanism of action. *Cancer Res.*, **48,** 1066-1071.

MILLS GB, MAY C, HILL M, CAMPBELL S, SHAW P AND MARKS A. (1990). Ascitic fluid from human ovarian cancer patients contains growth factors necessary for intraperitoneal growth of human ovarian adenocarcinoma cells. *J. Clin. Invest.*, **86,** 851-855.

MILNER BJ, ALLAN LA, ECCLES DM, KITCHENER HC, LEONARD RCF, KELLY KF, PARKIN DE AND HAITES NE. (1993). p53 mutation is a common genetic event in ovarian carcinoma. *Cancer Res.*, **53,** 2128-2132.

MORISHIGE K, KURACHI H, AMEMIYA K, FUJITA Y, YAMAMOTO T, MIYAKE A AND TANIZAWA O. (1993). Evidence for the involvement of transforming growth factor-α epidermal growth factor receptor autocrine growth mechanism in primary ovarian cancers. *Cancer Res.*, **51,** 5322-5328.

MOSMANN T. (1983). Rapid colorimetric assay for cellular growth and survival: Application to proliferation and cytotoxicity assays. *J. Immunol. Methods*, **65,** 55-63.

PELICCI G, LANFRANCONE L, GRIGNANI F, MCGLADE J, CAVALLO F, FORNI G, NICOLETTI I, GRIGNANI F, PAWSON T AND PELICCI PG. (1992). A novel transforming protein (SHC) with an SH2 domain is implicated in mitogenic signal transduction. *Cell*, **70,** 93-104.

RODRIGUEZ GC, BERCHUCK A, WHITAKER RS, SCHLOSSMAN D, CLARKE-PEARSON DL AND BAST RC Jr. (1991). Epidermal growth factor expression in normal ovarian epithelium and ovarian cancer. II. Relationship between receptor expression and response to epidermal growth factor. *Am. J. Obstet. Gynecol.*, **164,** 745-750.

SCAMBIA G, PANICI PB, BATTAGLIA F, FERRANDINA G, BAIOCCHI G, GREGGI S, DEVINCENZO R AND MANCUSO S. (1992). Significance of epidermal growth factor receptor in advanced ovarian cancer. *J. Clin. Oncol.*, **10,** 529-535.

SLAMON DJ, GODOLPHIN W, JONES LA, HOLT JA, WONG SG, KEITH DE, LEVIN WJ, STUART SG, UDOVE J, ULLRICH A AND PRESS MF. (1989). Studies of the HER/2-neu proto-oncogene in human breast and ovarian cancer. *Science*, **244,** 707-712.

STROMBERG K, COLLINS TJ, GORDON AW, JACKSON CL AND JOHNSON GR. (1992). Transforming growth factor-α acts as an autocrine growth factor in ovarian carcinoma cell lines. *Cancer Res.*, **52,** 341-347.

TENERIELLO MG, EBINA M, LINNOILA RI, HENRY M, NASH JD, PARK RC AND BIRRER MJ. (1993). p53 and Ki-*ras* gene mutations in epithelial ovarian neoplasms. *Cancer Res.*, **53,** 3103-3108.

ULLRICH A AND SCHLESSINGER J. (1990). Signal transduction by receptors with tyrosine kinase activity. *Cell*, **61,** 203-212.

VAN DE VIJVER MJ, KUMAR R AND MENDELSOHN J. (1991). Ligand-induced activation of A431 cell epidermal growth factor receptors occurs primarily by an autocrine pathway that acts upon receptors on the surface rather than intracellularly. *J. Biol. Chem.*, **266,** 7503-7508.

WU J, ROSSOMANDO AJ, HER J-H, DEL VECCHIO R, WEBER MJ AND STURGILL TW. (1991). Autophosphorylation *in vitro* of recombinant 42-kilodalton mitogen-activated protein kinase on tyrosine. *Proc. Natl Acad. Sci. USA*, **88,** 9508-9512.

ZHOU LI AND LEUNG BS. (1992). Growth regulation of ovarian cancer cells by epidermal growth factor and transforming growth factors-alpha and beta-1. *Biochim. Biophys. Acta*, **1080,** 130-136.


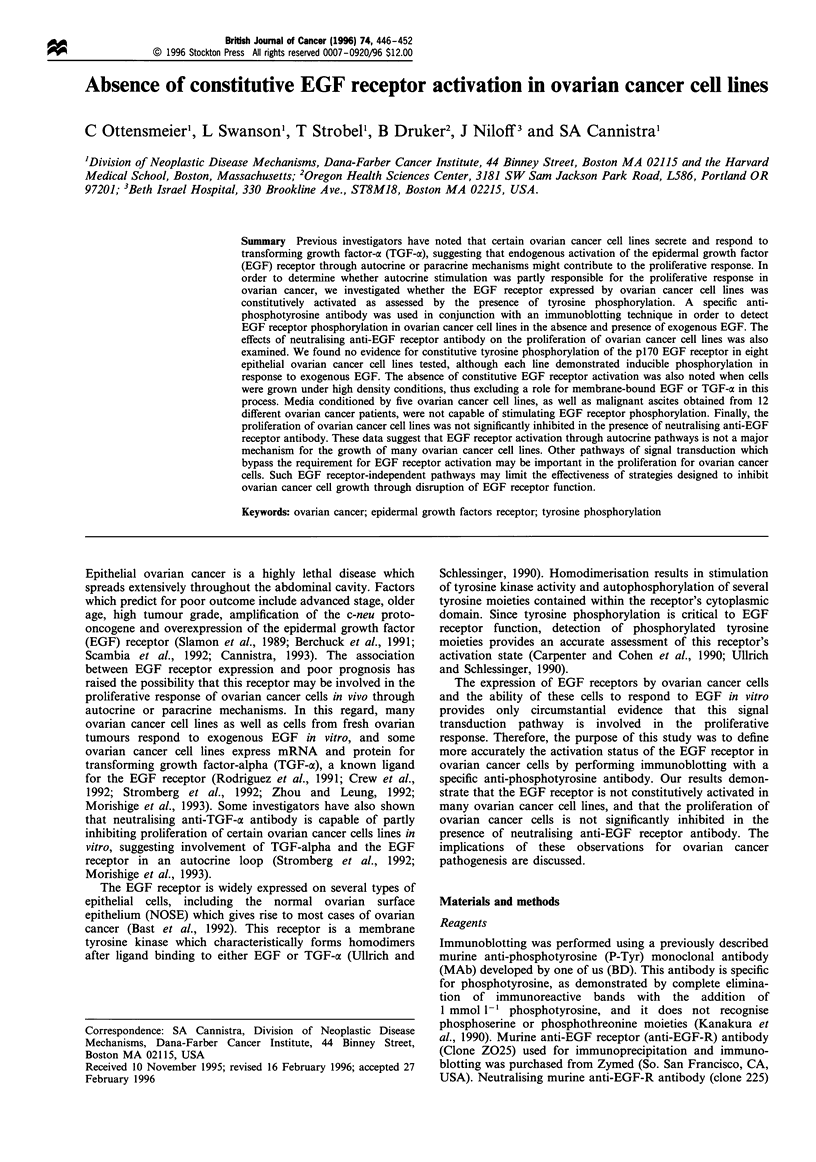

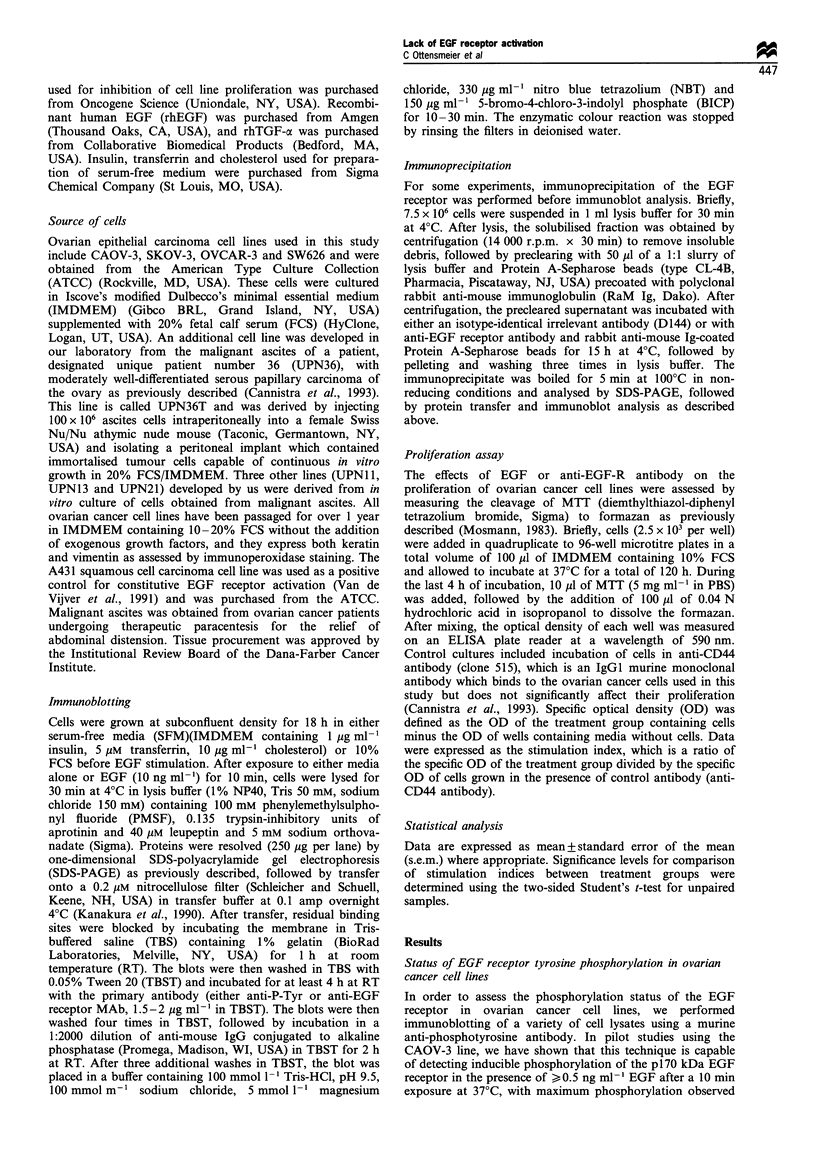

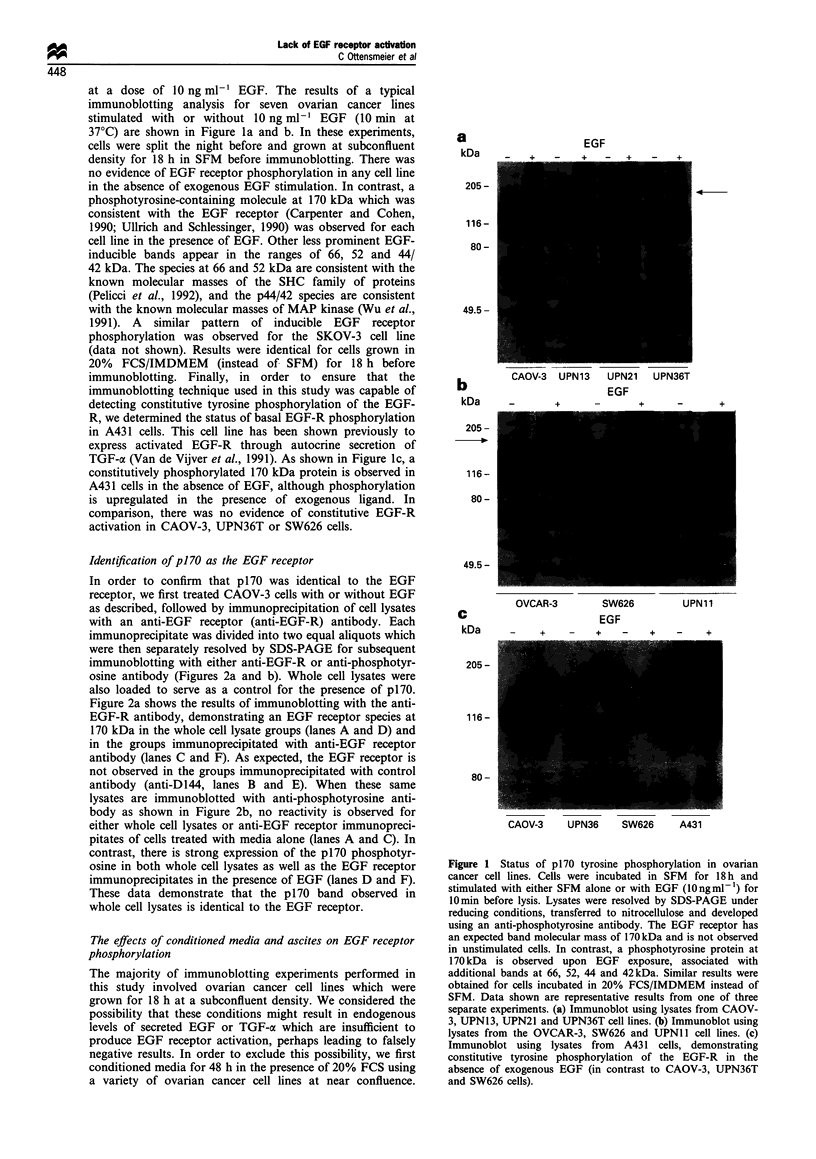

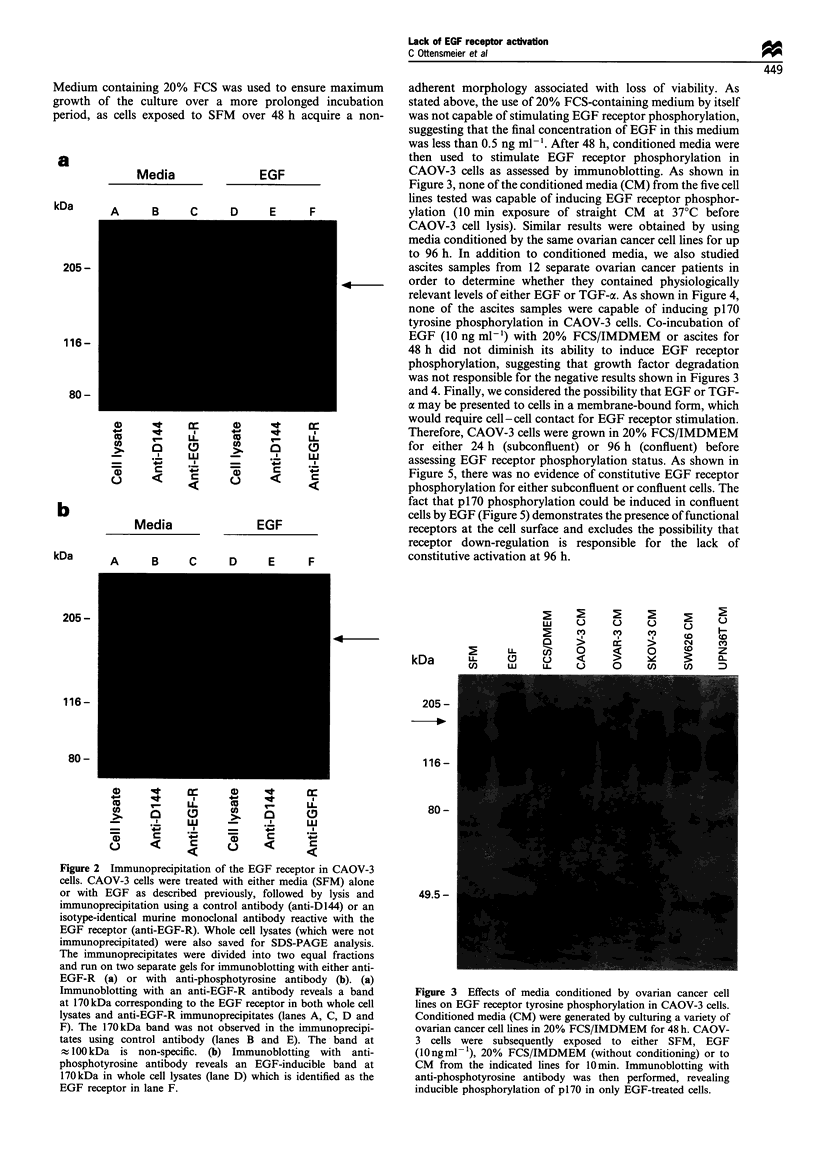

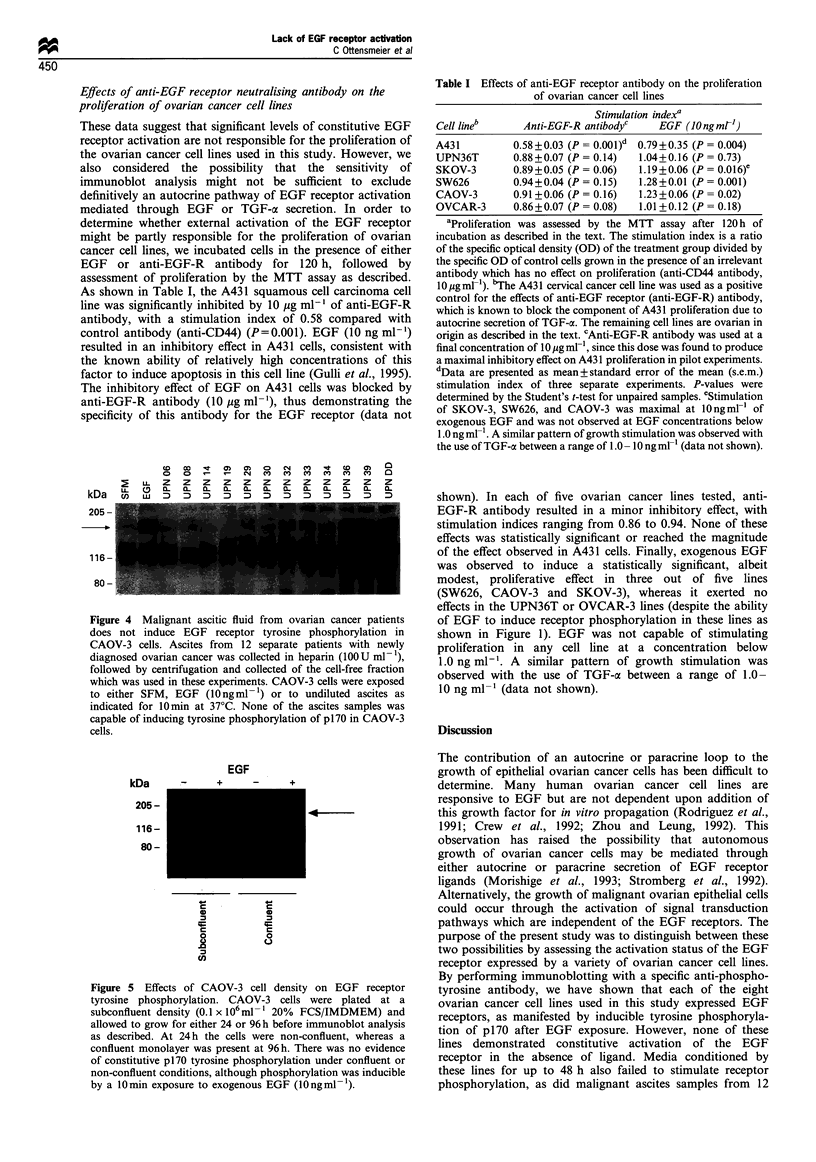

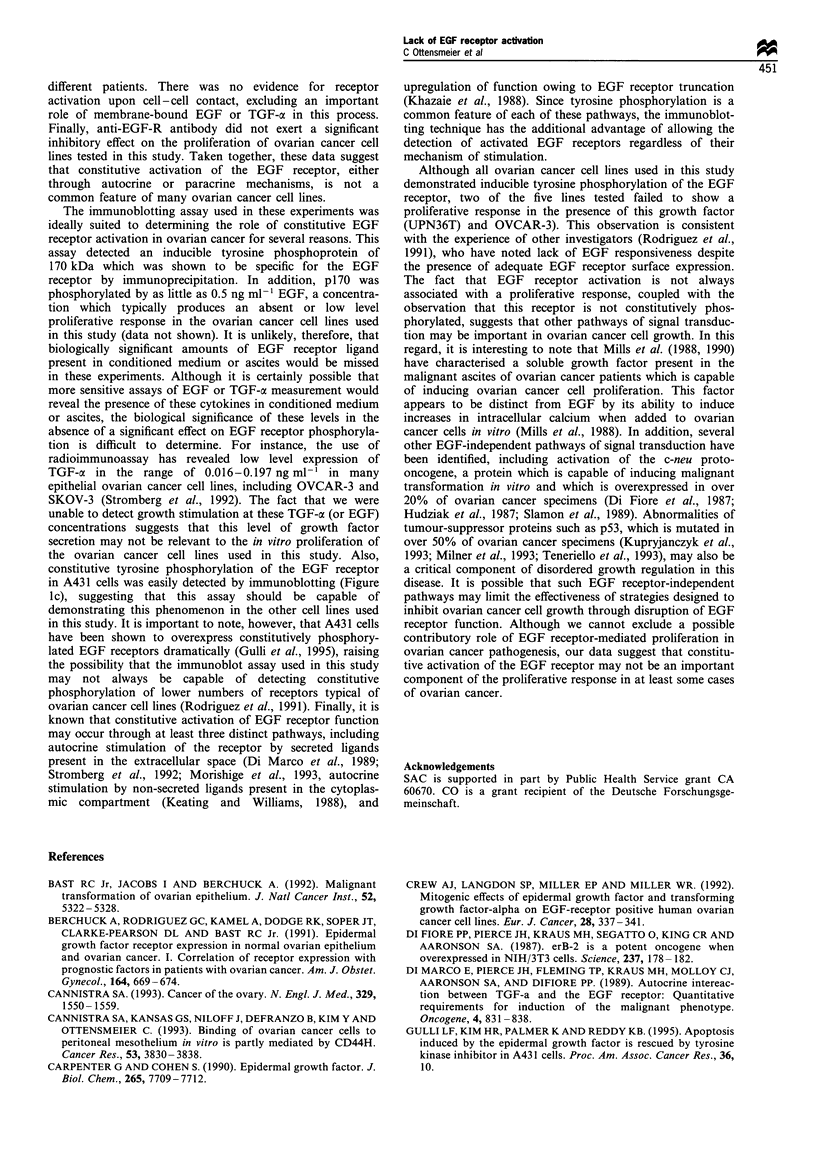

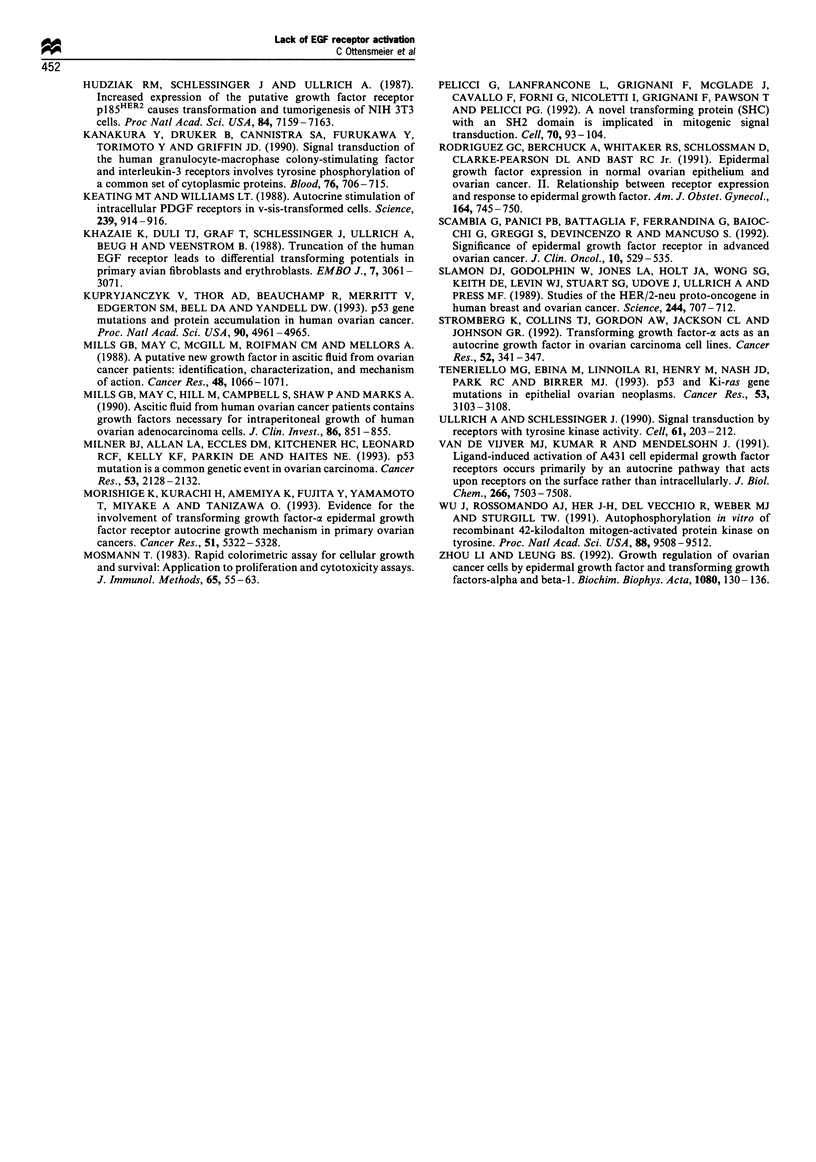


## References

[OCR_00892] Berchuck A., Rodriguez G. C., Kamel A., Dodge R. K., Soper J. T., Clarke-Pearson D. L., Bast R. C. (1991). Epidermal growth factor receptor expression in normal ovarian epithelium and ovarian cancer. I. Correlation of receptor expression with prognostic factors in patients with ovarian cancer.. Am J Obstet Gynecol.

[OCR_00898] Cannistra S. A. (1993). Cancer of the ovary.. N Engl J Med.

[OCR_00902] Cannistra S. A., Kansas G. S., Niloff J., DeFranzo B., Kim Y., Ottensmeier C. (1993). Binding of ovarian cancer cells to peritoneal mesothelium in vitro is partly mediated by CD44H.. Cancer Res.

[OCR_00910] Carpenter G., Cohen S. (1990). Epidermal growth factor.. J Biol Chem.

[OCR_00914] Crew A. J., Langdon S. P., Miller E. P., Miller W. R. (1992). Mitogenic effects of epidermal growth factor and transforming growth factor-alpha on EGF-receptor positive human ovarian carcinoma cell lines.. Eur J Cancer.

[OCR_00920] Di Fiore P. P., Pierce J. H., Kraus M. H., Segatto O., King C. R., Aaronson S. A. (1987). erbB-2 is a potent oncogene when overexpressed in NIH/3T3 cells.. Science.

[OCR_00925] Di Marco E., Pierce J. H., Fleming T. P., Kraus M. H., Molloy C. J., Aaronson S. A., Di Fiore P. P. (1989). Autocrine interaction between TGF alpha and the EGF-receptor: quantitative requirements for induction of the malignant phenotype.. Oncogene.

[OCR_00943] Hudziak R. M., Schlessinger J., Ullrich A. (1987). Increased expression of the putative growth factor receptor p185HER2 causes transformation and tumorigenesis of NIH 3T3 cells.. Proc Natl Acad Sci U S A.

[OCR_00950] Kanakura Y., Druker B., Cannistra S. A., Furukawa Y., Torimoto Y., Griffin J. D. (1990). Signal transduction of the human granulocyte-macrophage colony-stimulating factor and interleukin-3 receptors involves tyrosine phosphorylation of a common set of cytoplasmic proteins.. Blood.

[OCR_00956] Keating M. T., Williams L. T. (1988). Autocrine stimulation of intracellular PDGF receptors in v-sis-transformed cells.. Science.

[OCR_00962] Khazaie K., Dull T. J., Graf T., Schlessinger J., Ullrich A., Beug H., Vennström B. (1988). Truncation of the human EGF receptor leads to differential transforming potentials in primary avian fibroblasts and erythroblasts.. EMBO J.

[OCR_00968] Kupryjańczyk J., Thor A. D., Beauchamp R., Merritt V., Edgerton S. M., Bell D. A., Yandell D. W. (1993). p53 gene mutations and protein accumulation in human ovarian cancer.. Proc Natl Acad Sci U S A.

[OCR_00980] Mills G. B., May C., Hill M., Campbell S., Shaw P., Marks A. (1990). Ascitic fluid from human ovarian cancer patients contains growth factors necessary for intraperitoneal growth of human ovarian adenocarcinoma cells.. J Clin Invest.

[OCR_00974] Mills G. B., May C., McGill M., Roifman C. M., Mellors A. (1988). A putative new growth factor in ascitic fluid from ovarian cancer patients: identification, characterization, and mechanism of action.. Cancer Res.

[OCR_00986] Milner B. J., Allan L. A., Eccles D. M., Kitchener H. C., Leonard R. C., Kelly K. F., Parkin D. E., Haites N. E. (1993). p53 mutation is a common genetic event in ovarian carcinoma.. Cancer Res.

[OCR_00993] Morishige K., Kurachi H., Amemiya K., Fujita Y., Yamamoto T., Miyake A., Tanizawa O. (1991). Evidence for the involvement of transforming growth factor alpha and epidermal growth factor receptor autocrine growth mechanism in primary human ovarian cancers in vitro.. Cancer Res.

[OCR_00999] Mosmann T. (1983). Rapid colorimetric assay for cellular growth and survival: application to proliferation and cytotoxicity assays.. J Immunol Methods.

[OCR_01006] Pelicci G., Lanfrancone L., Grignani F., McGlade J., Cavallo F., Forni G., Nicoletti I., Grignani F., Pawson T., Pelicci P. G. (1992). A novel transforming protein (SHC) with an SH2 domain is implicated in mitogenic signal transduction.. Cell.

[OCR_01009] Rodriguez G. C., Berchuck A., Whitaker R. S., Schlossman D., Clarke-Pearson D. L., Bast R. C. (1991). Epidermal growth factor receptor expression in normal ovarian epithelium and ovarian cancer. II. Relationship between receptor expression and response to epidermal growth factor.. Am J Obstet Gynecol.

[OCR_01019] Scambia G., Benedetti Panici P., Battaglia F., Ferrandina G., Baiocchi G., Greggi S., De Vincenzo R., Mancuso S. (1992). Significance of epidermal growth factor receptor in advanced ovarian cancer.. J Clin Oncol.

[OCR_01025] Slamon D. J., Godolphin W., Jones L. A., Holt J. A., Wong S. G., Keith D. E., Levin W. J., Stuart S. G., Udove J., Ullrich A. (1989). Studies of the HER-2/neu proto-oncogene in human breast and ovarian cancer.. Science.

[OCR_01029] Stromberg K., Collins T. J., Gordon A. W., Jackson C. L., Johnson G. R. (1992). Transforming growth factor-alpha acts as an autocrine growth factor in ovarian carcinoma cell lines.. Cancer Res.

[OCR_01038] Teneriello M. G., Ebina M., Linnoila R. I., Henry M., Nash J. D., Park R. C., Birrer M. J. (1993). p53 and Ki-ras gene mutations in epithelial ovarian neoplasms.. Cancer Res.

[OCR_01043] Ullrich A., Schlessinger J. (1990). Signal transduction by receptors with tyrosine kinase activity.. Cell.

[OCR_01047] Van de Vijver M. J., Kumar R., Mendelsohn J. (1991). Ligand-induced activation of A431 cell epidermal growth factor receptors occurs primarily by an autocrine pathway that acts upon receptors on the surface rather than intracellularly.. J Biol Chem.

[OCR_01052] Wu J., Rossomando A. J., Her J. H., Del Vecchio R., Weber M. J., Sturgill T. W. (1991). Autophosphorylation in vitro of recombinant 42-kilodalton mitogen-activated protein kinase on tyrosine.. Proc Natl Acad Sci U S A.

[OCR_01058] Zhou L., Leung B. S. (1992). Growth regulation of ovarian cancer cells by epidermal growth factor and transforming growth factors alpha and beta 1.. Biochim Biophys Acta.

